# A bibliometric analysis of programmed cell death in acute lung injury/acute respiratory distress syndrome from 2000 to 2022

**DOI:** 10.1016/j.heliyon.2023.e19759

**Published:** 2023-09-01

**Authors:** Enyao Huang, Li Gao, Ruiyu Yu, Keying Xu, Lihong Wang

**Affiliations:** a Department of Pathophysiology, Medical College of Southeast University, Nanjing, 210009, China; b Jiangsu Provincial Key Laboratory of Critical Care Medicine, Nanjing, 210009, China

**Keywords:** Bibliometrics, Programmed cell death, Acute lung injury, Acute respiratory distress, Apoptosis, Ferroptosis, Pyroptosis

## Abstract

Acute lung injury (ALI) is a prevalent critical disorder that disrupts the body's homeostasis in patients. The progression from ALI to acute respiratory distress syndrome (ARDS) is often accompanied by programmed cell death (PCD). However, there has been a lack of systematic research and comprehensive analysis on the role of different types of PCD in ALI/ARDS. This study aims to analyze the research status, trends, research hotspots, and compare the contribution of publications from different countries, institutions, journals and authors in the field of PCD in ALI/ARDS using bibliometric analysis. We collected publications regard to PCD and ALI/ARDS from Web of Science during 2000–2022. VOSviewer, Citespace, Scimago Graphica, Pajek, and GraphPad Prism 9.0 software were used for further analyzed and visualized. We identified a total of 3495 publications. The number of publications has increased since the beginning of the new century. China produced the most publications (1965), while the United States ranks first in the number of citations (40141). Shanghai Jiao Tong University and American Journal of Physiology-Lung Cellular and Molecular Physiology were the most prolific institution and journal, respectively. Wang, Ping has published most papers (23) while publications from Lee, Pj have most citations (2016). In terms of keywords, “apoptosis” and “inflammation” are the most frequently occurring, but there has been a recent shift from “apoptosis” and “autophagy” to “necroptosis”, “pyroptosis”, and “ferroptosis”. Additionally, COVID-19 and long noncoding RNA (lncRNA) have become research hotspots in recent years. In conclusion, this bibliometric analysis reveals the research directions and frontier hotspots of PCD in ALI/ARDS. China and the United States have made important contributions to the development of this field. The research hotspots have recently focused on necroptosis, pyroptosis, ferroptosiss, COVID-19 and lncRNA.

## 1 Introduction

Acute lung injury (ALI) is an acute, progressive hypoxic respiratory failure that eventually evolves into acute respiratory distress syndrome (ARDS). ALI/ARDS is a common serious disorder of homeostasis in the body, which can easily lead to the death of patients [1]. Despite significant efforts by researchers and clinicians, the pathogenesis of acute lung injury remains unclear [2]. Alveolar epithelial cells and vascular endothelial cell injury and death are central processes in the development of ALI [3]. Hence, investigating the potential role of programmed cell death (PCD), an important category of cell death, in ALI could have significant prospects for improving understanding and treatment of this condition.

Since the initial definition of PCD, the types of cell death it encompasses have continuously expanded. Currently, the most widely studied types of PCD include apoptosis, autophagy, necroptosis, pyroptosis, and ferroptosis [4]. Apoptosis is a highly regulated form of cell death triggered by various pathways, with caspase activation playing a central role [5]. Autophagy is a self-lysis process in cells characterized by the formation of autophagosomes, which serve as intracellular degradation system for maintaining organelle turnover [6]. The discovery of necroptosis, which is activated by extracellular stimuli and known to cause inflammation and cell death, challenges the traditional concept of necrosis [7]. Pyroptosis is a genetically encoded, gasdermin-dependent cell lysis pathway and is a primary cellular response to potentially harmful insults [8]. Ferroptosis is a novel type of cell death caused by iron overload and ROS-dependent accumulation of lipid peroxides, characterized by the reduction of the core enzyme GPX4 in the regulation of the antioxidant system (glutathione system) [9]. These five types of PCD have been extensively studied by researchers over the years, and numerous studies have shown their importance in the development and prognosis of ALI/ARDS [[10], [11], [12], [13], [14]].

The pathophysiological mechanism of ALI/ARDS has been extensively investigated, with PCD being recognized as a significant contributor. However, there remains a lack of systematic summarization and analysis of academic research specifically focusing on PCD in this field. Several key questions regarding the research trends in PCD, the evolution of research hotspots, and the collaborations among researchers and research institutions worldwide remain unanswered. Bibliometrics, as an interdisciplinary field of quantitative analysis, is widely utilized to investigate research trends, hotspots, and future prospects in the fields of medicine and biology. Leveraging bibliometric methods, this study aims to quantitatively analyze and visually represent the interrelationships among 3495 academic articles within this field since the beginning of the new century. By doing so, this study provides a comprehensive summary of the research efforts made by scholars in this field over the past two decades. Additionally, it presents an analysis of the historical evolution and current state of research within this field, serving as a valuable resource for researchers seeking to conduct in-depth investigations in related areas. Furthermore, this study offers relevant predictions for future research directions in the field [15,16].

## 2 Research methods

### 2.1 The data source

The literatures in our study were retrieved and downloaded from the Science Citation Index-Expanded (SCI-E) of Web of Science Core Collection (WoSCC), developed by Thomson Scientific. Since its creation, the database has been accepted by researchers and widely praised for its high-quality digital literature resources. It is considered to be a suitable database for bibliometric research [17].

### 2.2 Search strategies

The primary objective of this study is to investigate the role of PCD in ALI/ARDS, so the search keywords are limited to PCD and ALI/ARDS. Consequently, the search keywords were specifically limited to PCD and ALI/ARDS. The inclusion criteria for retrieved academic publications required them to satisfy both PCD and ALI/ARDS simultaneously, resulting in a logical “and” relationship in the search formula. Considering that ALI/ARDS may be referred to by their full name or abbreviation, the retrieved publications were expected to contain either one of these terms. Additionally, based on preliminary research, this study narrowed down the focus of PCD to the top five types: apoptosis, autophagy, necroptosis, pyroptosis, and ferroptosis. Consequently, retrieved publications only needed to encompass at least one of these five PCD types. Furthermore, the search for relevant literature was restricted to the title, abstract, and keyword sections. If a publication met the search criteria in these three parts, it was included in the target database for this study. The literature retrieval date of this study is controlled from 2000.1.1 to 2022.12.21, to avoid errors caused by the rapid update of the literature database. The search terms were set as follows: TS= ((Apoptosis) OR (Necroptosis) OR (Ferroptosis) OR (Autophagy) OR (Pyroptosis)) AND TS= ((ARDS) OR (ALI) OR (Acute lung disease) OR (Acute respiratory disease syndrome)).

### 2.3 Data collection and cleaning

**Fig. 1:**
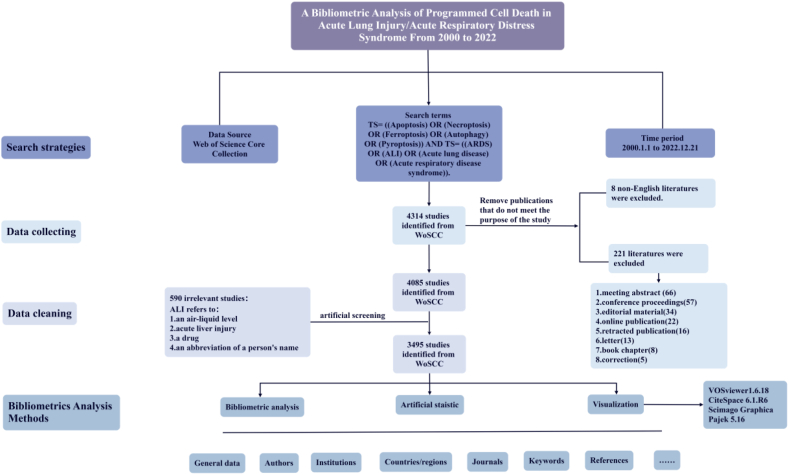
Flowchart of the data preparation process.

After effective literature retrieval, the original data extracted from the selected literature include: paper name, author, author's affiliation, author's country and region, journal name, publishing time, keywords, references, and citation times and so on. Due to the different format requirements of different journals for paper references and the different abbreviations of authors' names, there may be some deviations in the extracted data, but most of the original data are reliable. After collecting the original data of the literature, we clean the data such as merging synonyms in the original data and eliminating misspelled words, to obtain reliable original data. Due to the deviations in the retrieval, including the large scope of the search terms, some articles that have been withdrawn and so on. For example, ALI can refer to more than acute lung injury. ALI can also refer to an air-liquid level, acute liver injury, a drug, or an abbreviation of a person's name. Literature irrelevant to this study was manually screened out. Finally, the number of valid documents was 3495. The process of document retrieval and data cleaning is shown in Fig. 1.

### 2.4 Bibliometrics analysis methods

The birth of bibliometrics can be traced back to 1917 when researchers used quantitative methods to study literature. The concept of bibliometrics was first proposed in 1969. Bibliometrics is an interdisciplinary science that quantitatively analyzes all knowledge carriers through mathematical and statistical methods [18]. The bibliographic data is studied through the analysis method of bibliometrics, and the results are presented through visualization software.

Bibliometrics makes a quantitative analysis of the relevant indicators of academic publications retrieved from the database, such as the number of citations, the author's affiliation. By utilizing the data obtained from the analysis, we have employed visualizations to construct knowledge maps that include clustering hotspots, institutional rankings, and high-cited article rankings. These knowledge maps serve the purpose of exploring scientific research gaps, identifying frontier hotspots, and speculating on the development trends within the field. Such a data analysis method for mining related research in the field can give researchers more direct and intuitive results. It can help researchers who want to study in a certain field to find scientific research ideas and partners to save time.

VOSviewer1.6.18 is one of the many scientific knowledge map software, which realizes the drawing of scientific knowledge map through the relationship construction and visual analysis between data units, and shows the structure, evolution and cooperation of knowledge field. Its distinctive feature is that it has a unique visual effect style different from CiteSpace [19].

CiteSpace 6.1.R6 is citation visualization analysis software that focuses on analyzing the potential knowledge contained in the WoSCC. It is gradually developed in the context of scientometrics and data visualization. After decades of development, it has been possible to present the structure, laws, and distribution of scientific knowledge through visual means, and to display the laws behind complex data very intuitively [20].

Scimago Graphica is data analysis software that does not require any formulas, just drag, and drop to generate various charts. This study uses the software to beautify some of the excavated data results [21]

Pajek 5.16 is a large-scale complex network analysis tool. It is a powerful tool for studying various complex nonlinear networks that currently exist. It is used for analysis and visualization operations of large networks with thousands or even millions of nodes. At the same time, this study uses the software to optimize the visual graphics presented by VOSviewer [22].

This study will present accurate and beautiful visual analysis results by combining the above software. Meanwhile, in this study, some results are used GraphPad Prism 9.0 for statistical analysis.

## 3 Results

### 3.1 The overview and trend of global publications

**Fig. 2:**
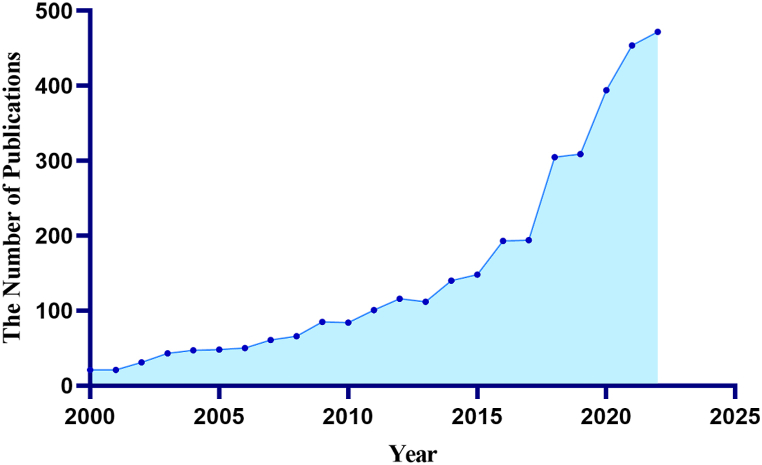
The number of publications on the role of PCD in ALI/ARDS from 2000 to 2022.

From 2000 to 2022, there were a total of 3495 publications on the role of programmed cell death in ALI/ARDS. Fig. 2 depicts the overall growth trend of annual publications, which can be divided into two stages: 2000–2014 is a steady growth stage, and 2015–2022 is a rapid growth stage. It showed a significant growth trend in 2018 and 2020. In recent years, the epidemic has spread globally. It mainly attacks the lungs of patients which is easy to cause acute lung injury and even death of patients, for which reason, the publications in this field continue to grow, and more and more scholars have obtained remarkable scientific achievements in this field, which promotes further development in this field [23].

### 3.2 The contribution of countries to global publications

**Fig. 3:**
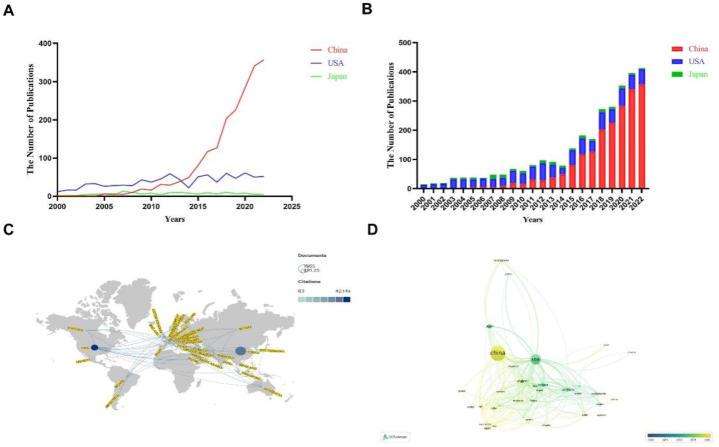
The mapping on countries publishing researches on the role of PCD in ALI. (A and B) The number of publications by China, the United States, and Japan from 2000 to 2022. (C) World map of publications. Note: The size of the circle indicates the number of documents published. The color of the circle indicates the number of citations. The connection between nodes indicates cooperation between countries. (D) The cooperation diagram of countries involved in the role of PCD in ALI/ARDS in the publications. Note: The size of the circle indicates the number of papers published in the country. The color of the circle indicates the year of publication. The connection between the circle nodes represents the cooperation between countries.

By 2022, there are 3495 publications on the role of programmed cell death in ALI/ARDS worldwide. Among them, the country with the most publications is China, with 1965 publications, accounting for 56.22%, which indicates that Chinese researchers have done a lot of studies in the field of the role of PCD in ALI/ARDS. The second is the United States, with 903 publications, accounting for 25.84%. The third is Japan, with 151 publications, accounting for 4.32%. But in terms of total citations, the United States ranks first (Table 1). It can be seen from Fig. 3A and B that before 2008, the global literature on the role of PCD in ALI/ARDS was mainly published in the United States, while publications in China and Japan were fewer. Over time, starting in 2005, China and Japan began to publish documents in this field. Since 2009, the number of publications in China has gradually increased. The number of publications in the United States and Japan basically unchanged, especially in Japan. Compared with the United States and Japan, the number of publications in China shows a clear growth curve (Fig. 3A). In 2014, the number of papers published by China in this field began to exceed that of the United States. Especially in 2018 and 2020, the number of publications in China has increased significantly. The countries in this field are placed on the world map, and the countries are set as circle nodes (Fig. 3C and D). We can find that China has the largest number of publications, and the United States has the largest number of citations. China and the United States, especially the United States, have close cooperation with surrounding countries. We use VOSviewer to make a time relationship figure of each country and their publications, and set the country as a circle node. According to the figure, from 2000 to 2022, the center of publishing papers in this field gradually shifts from the United States to China. It is hoped that in the future, China will publish more and better publications to contribute to the development in this field.

**Table 1:** The top 10 countries with the most publications.

Rank	Country	Documents	Count (%)	Citations
1	China	1965	56.22	31145
2	USA	903	25.84	40141
3	Japan	151	4.32	4890
4	Germany	122	3.49	4396
5	Canada	119	3.40	6735
6	England	96	2.75	4404
7	Italy	74	2.12	3921
8	South Korea	68	1.95	1481
9	India	58	1.66	862
10	Brazil	55	1.57	1893

### 3.3 Contribution of institutions on the role of programmed cell death in acute lung injury

Table 2 lists the top 10 institutions with the highest Np of the role of PCD in ALI. The top three institutions with the highest Np were Shanghai Jiao Tong University, Fudan University and China Med University. Surprisingly, nine of the top 10 productive institutions are from China. The only one left is the University of Toronto from Canada. Although its number of publications is not in the top five, the citations were more than twice that of any other institution.

**Table 2:** The top 10 productive institutions.

Rank	Organization	Documents	Citations
1	Shanghai Jiao Tong University	108	2049
2	Fudan University	75	1408
3	China Med University	72	1100
4	Wuhan University	65	792
5	Tongji University	64	1236
6	Wenzhou Med University	61	959
7	Nanjing Med University	60	583
8	Huazhong University Sci&Technology	56	944
9	University of Toronto	54	4044
10	Southern Med University	49	684

**Fig. 4:**
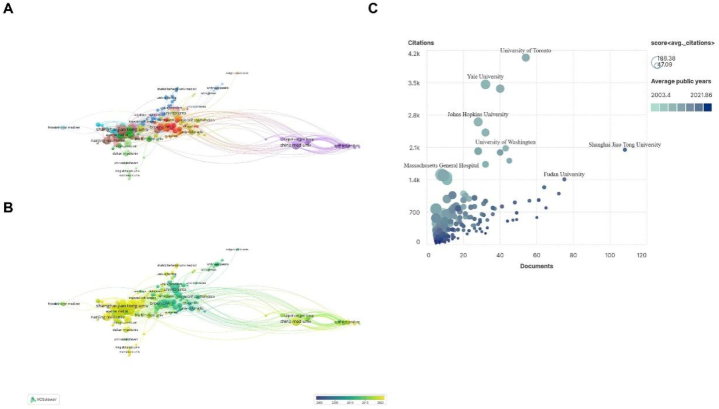
The mapping of institutions publishing research on the role of PCD in ALI. (A) The cooperation map of productive institutions. Note: The size of the node represents the frequency of occurrence. The color of the node represents the different clusters. The thickness of the lines represents the link strength. (B) Visualization of productive institutions according to the APY. Note: The size of the node represents the frequency of occurrence. The color of the node represents the year. (C) Coordinate map of productive institutions. Note: The abscissa represents the published documents, and the ordinate represents the number of citations. The shade of the color represents the average number of citations. The darker the color, the later the document is published.

Fig. 4 shows that in recent years, the focus of development has gradually shifted from the University of Toronto, Brown University and other universities to Fudan University and Shanghai Jiao Tong University in China (Fig. 4A and B). We can see that the latter has a larger number of publications, but the number of citations is not very considerable. In comparison, although there were no many publications from the University of Toronto and Yale University, they had high citations (Fig. 4C).

### 3.4 Analysis of authors on the role of programmed cell death in acute lung injury

**Fig. 5:**
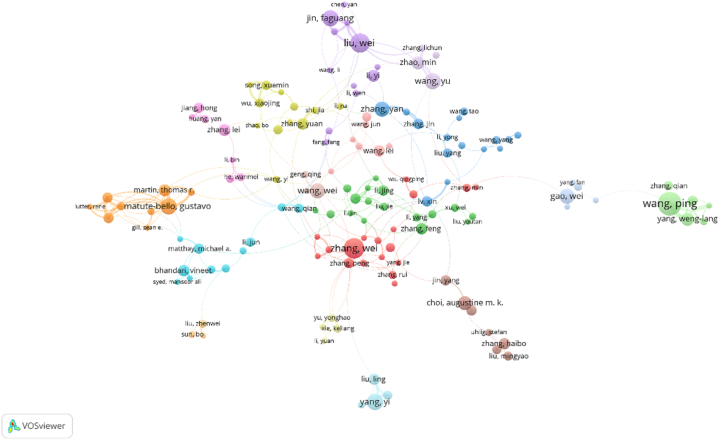
Author visualization of VOSviewer. Note: The circle represents the number of publications by the author. The lines between nodes represent the collaboration between authors, and the collaboration graph indicates that the author's collaboration does exist in this field. Color represents clustering, and the authors of the same color indicates that the fields of their publications belong to a cluster, and there are many connections between them.

Table 3 sorts the authors according to the number of publications. We can see that Wang, Ping (23), comes the first, followed by Ayala, Alfred (21), the third is Zhang,Wei (20). Table 4 ranks the authors according to the number of citations. It can be seen that Lee, Pi's publications were cited the most times (2016), followed by Martin,Tr (1276), and the third was Garcia,Jgn (1118). The above three authors have been cited more than 1000 times, but their number of publications is less than 10. The result means they have published influential publications and made great contributions to the role of PCD in ALI/ARDS. We screened authors with less than 6 publications and low cooperation between each other, and finally retained 144 authors for analysis. We use the software VOSviewer and make a cooperative clustering figure (Fig. 5). We find that Wang Ping has the most publications, but his cooperation with other authors is relatively less. Zhang Wei has many publications and more cooperation with other authors.

**Table 3:** The top 10 authors related to the role of PCD in ALI/ARDS ranked by number of publications.

Rank	Author	Documents	Citations	Average Citations
1	Lee, Pj	6	2016	336
2	Martin, Tr	10	1276	127.6
3	Garcia, Jgn	6	1118	186.3
4	Matthay, Michael A	10	926	92.6
5	Choi, Augustine M.K.	14	854	61
6	Matute-Bello, G	8	815	101.9
7	Ayala, Alfred	21	806	38.4
8	Hashimoto, S	5	726	145.2
9	Tuder, Rubin M.	6	719	119.8
10	Wang, Ping	23	709	30.8

**Table 4:** The top 10 authors related to the role of PCD in ALI/ARDS ranked by number of citations.

Rank	Author	Documents	Citations	Average Citations
1	Wang, Ping	23	709	30.8
2	Ayala, Alfred	21	806	38.4
3	Zhang, Wei	20	179	8.9
4	Liu, Wei	19	238	12.5
5	Kolliputi, Narasaiah	18	554	30.8
6	Chung, Chun-Shiang	17	709	41.7
7	Matute-Bello, Gustavo	16	544	34
8	Yang, Yi	16	457	28.6
9	Wang, Yu	16	290	18.1
10	Jin, Faguang	16	152	9.5

### 3.5 Analysis of journals publishing researches on the role of programmed cell death in acute lung injury

**Fig. 6:**
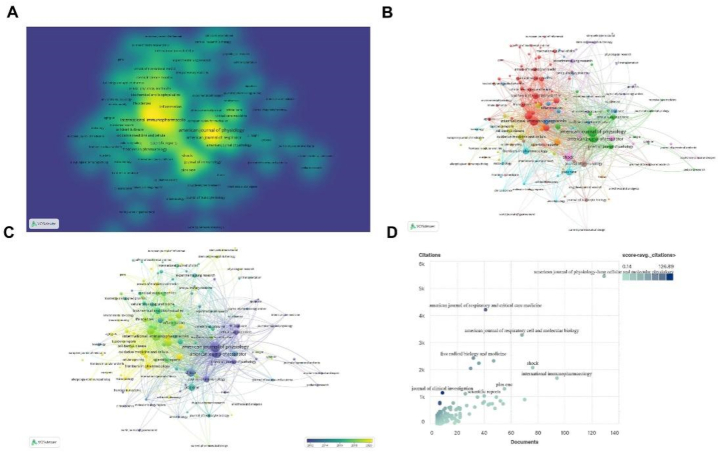
The mapping of journals publishing research on the role of PCD in ALI. (A) Density visualization of productive journals. Note: The size and color of the word represent the frequency of the journals. The larger the word, the brighter the color, the higher the frequency of occurrence. (B) The cooperation map of productive journals. Note: The journals involved were divided into four clusters by different colors: cluster 1: green, cluster 2: red, cluster 3: blue, cluster 4: purple. Note: The size of the node represents the frequency of occurrence. The color of the node represents the different clusters. The thickness of the lines represents the link strength. (C) Visualization of productive journals. Note: The size of the node represents the frequency of occurrence. The color of the node represents the year. (D) Coordinate map of production journals. Note: The abscissa represents the published documents, and the ordinate represents the number of citations. The shade of the color represents the average number of citations.

In the hotspot map, we can see that the highest frequency is “American Journal of Physiology-Lung Cellular and Molecular Physiology”, “International Immunopharmacology”, “shock” (Fig. 6A). Time view displays the focus of journals that frequently published documents on related themes shifted from “American Journal of Physiology” to “International Immunopharmacology” (Fig. 6B and C). However, the coordinate diagram shows that although these two rank the top in the number of publications, their average citations are not high. “Journal of clinical investigation” has a significant average citation despite the smaller number of publications (Fig. 6D).

### 3.6 Dual-map overlay

**Fig. 7:**
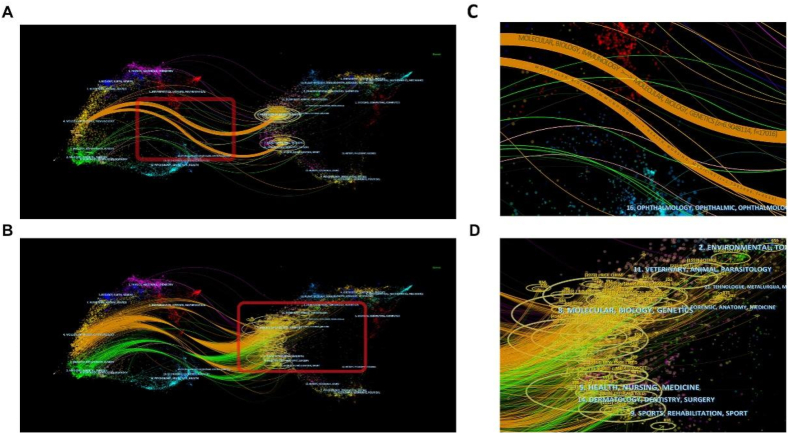
The dual-map overlay using z-score function. (A) The co-citation links of the cited journals. (B) The citing and cited trajectory from 2000 to 2022. (C and D) The partial enlargement of the trajectory. Note: Different colors represent different discipline categories. The thicker the connection between disciplines, the stronger the citation relationship and vice versa.

The left and right sides of the dual-map overlay layer represent the citing and cited journals, and are connected to each other through citation waves (Fig. 7A and C). The citing side can be considered as the research frontier, and the cited side can be considered as the basis of their research [24]. In a dual-map overlay on the theme of the role of PCD in ALI, the disciplines “Molecular, Biology, Immunology” and “Medicine, Medical, Clinical” are based on “Molecular, Biology, Genetics” and “Health, Nursing, Medicine” (Fig. 7B and D). In addition, “Dermatology, Dentistry, Surgery” and “Veterinary, Animal, Parasitology” are also referenced more (Fig. 7C).

### 3.7 Highly cited publications

Among the 3495 publications, we selected 10 publications with the strongest citation burst with the role of PCD in ALI/ARDS (Table 5). We can see that there are two of the publications from “AMER PHYSIOLOGICAL SOC”, two publications from “ELSEVIER SCIENCE INC”. Among them, the impact factor of the first publication from NATURE MEDICINE is the highest, reaching 87.244. And the number of citations is also the highest (Regulation of lung injury and repair by Toll-like receptors and hyaluronan).

**Table 5:** The top 10 publications with the most citiations

Rank	Name	Averages	Total Cites	Journal	IF	Journal Citation Indicator
1	**Regulation of lung injury and repair by Toll-like receptors and hyaluronan**	56.37	1071	NATURE MEDICINE	87.244	13
2	**Mechanisms of bacterial lipopolysaccharide-induced endothelial apoptosis**	12.29	258	AMER PHYSIOLOGICAL SOC	6.011	1.44
3	**Fas/FasL-dependent apoptosis of alveolar cells after lipopolysaccharide-induced lung injury in mice**	10.91	251	AMER THORACIC SOC	30.525	4.14
4	**Caspase-11-mediated endothelial pyroptosis underlies endotoxemia-induced lung injury**	30.43	213	AMER SOC CLINICAL INVESTIGATION INC	19.477	3.93
5	**PAMAM Nanoparticles Promote Acute Lung Injury by Inducing Autophagic Cell Death through the Akt-TSC2-mTOR Signaling Pathway**	13.27	199	OXFORD UNIV PRESS	8.185	0.74
6	**Fas (CD95) induces alveolar epithelial cell apoptosis in vivo - Implications for acute pulmonary inflammation**	8.39	193	ELSEVIER SCIENCE INC	5.77	1.53
7	**Protection from lethal apoptosis in lipopolysaccharide-induced acute lung injury in mice by a caspase inhibitor**	7.63	183	ELSEVIER SCIENCE INC	5.77	1.53
8	**NLRP1-Dependent Pyroptosis Leads to Acute Lung Injury and Morbidity in Mice**	14	168	AMER ASSOC IMMUNOLOGISTS	5.43	0.92
9	**Acute lung injury and cell death: how many ways can cells die?**	10.38	166	AMER PHYSIOLOGICAL SOC	6.011	1.44
10	**The role of apoptosis in acute lung injury**	7.62	160	LIPPINCOTT WILLIAMS & WILKINS	9.296	1.47

### 3.8 Analysis of Co-citation publications on the role of programmed cell death in acute lung injury

**Fig. 8:**
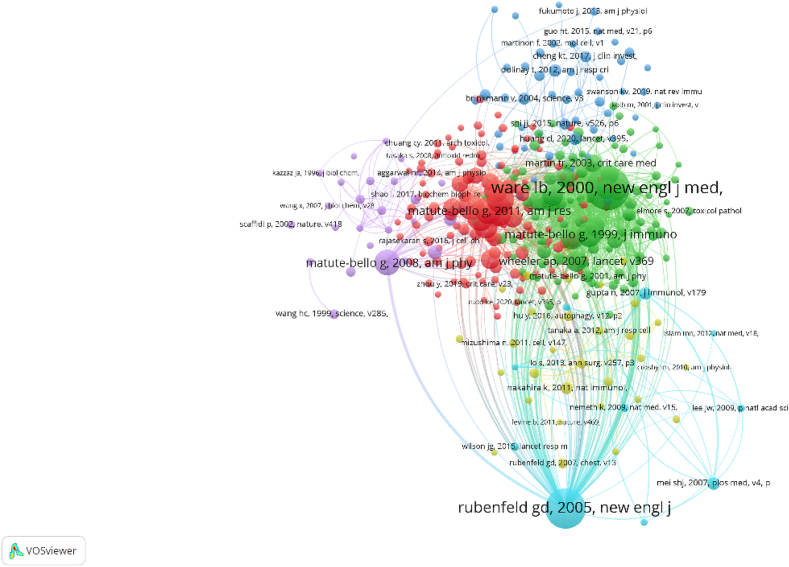
The co-cited references mapping of publications related to PCD in ALI/ARDS from 2000 to 2022.Note: The size of the node circle represents the number of citations in the publications. The color of the circle represents clustering. The connection between nodes represents the common reference relationship.

We imported the relevant publications on the role of PCD in ALI/ARDS into VOSviewer to make a visual map of the co-cited literature (Fig. 8). We can see that most of the highly co-cited literature was published earlier. Meanwhile, publication written by Rubenfeld gd in 2005 was cited by most publications. However, the number of citations of publications clustered with it is not high.

### 3.9 Analysis of keywords on the role of programmed cell death in acute lung injury

#### 3.9.1 Co-occurrence analysis of keywords clusters

**Fig. 9:**
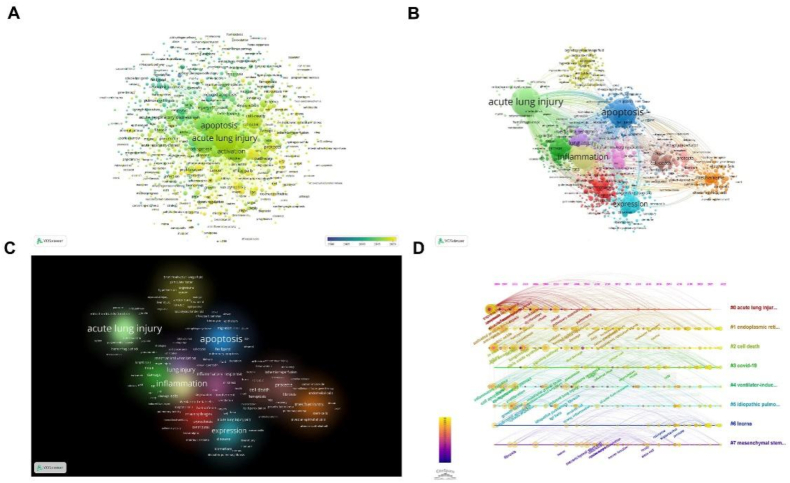
The mapping on keywords of the role of PCD in ALI and the timeline view of co-citation clusters. (A) Visualization of keywords 1. Note: The size of the node represents the frequency of occurrence. The color of the node represents the time when the keyword appeared. (B) Visualization of keywords 2. Note: The size of the node represents the frequency of occurrence. Note: The color of the node represents the different clusters. The thickness of the lines represents the link strength. (C) Density visualization of keywords. The size and color of the word represent the frequency of the keyword. Note: The larger the word, the brighter the color, the higher the frequency of occurrence. (D) The timeline view of co-citation clusters with their cluster-labels on the right.

Keywords are the refinement of the theme of an article. Keyword co-occurrence analysis can easily obtain various research directions in a field. We use VOSviewer to visualize popular topics in this field (Fig. 9A). The most common keywords are “apoptosis”, “inflammation”, “expression” and “mechanisms”, which indicates that basic research on the role of PCD in ALI is fruitful in terms of cell death mechanisms (Fig. 9B and C). Meanwhile, we can get that in recent years, the focus of PCD in this area has shifted from “apoptosis” to “pyroptosis”, “Necroptosis”, “Ferroptosis” and “Autophagy” (Fig. 9A). The phenomenon suggests that this field continues to open more research directions and hotspots.

Fig. 9D shows that apoptosis and ALI have the most citation bursts. The relationship between apoptosis and ALI has been extensively studied by researchers in earlier years. However, in recent years, the research focus has gradually shifted to the effect of LncRNA on ALI by regulating PCD. At the same time, the research about this field has been a hotspot due to the impact of the COVID-19 on human health in the past three years.

#### 3.9.2 Burst detection

**Fig. 10:**
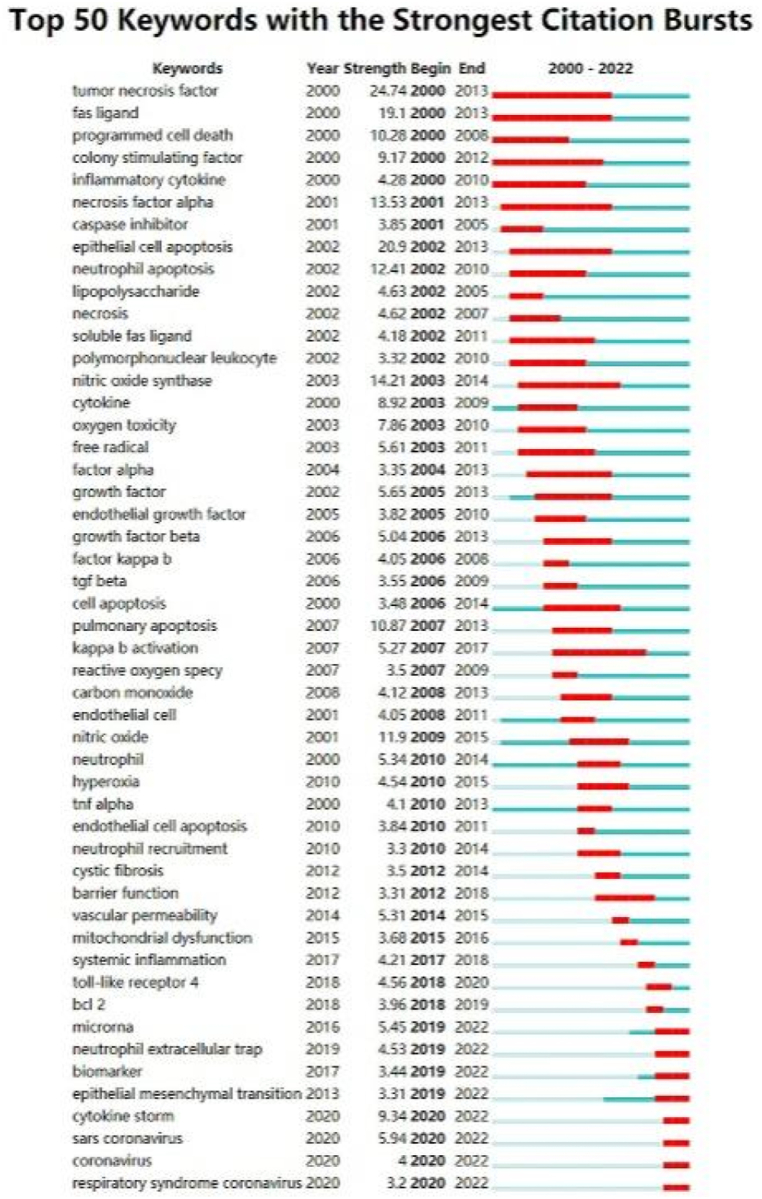
The top 50 keywords with the strongest citation bursts. Note: The blue line denotes the timeline, and the red sections denote the burst interval, respectively, showing the beginning and end of the year, and the burst duration.

Keyword burst detection refers to the detection of keywords with high frequency in a certain period of time, which helps researchers analyze the evolution of procedural death research [25]. In our study, the time period is 2000–2022. As shown in Fig. 10, we identified 50 keywords with the strongest citation prominence. We found that the most frequently cited keyword was tumor necrosis factor (Strength = 24.74), followed by epithelial cell apoptosis (Strength = 20.9) and Fas ligand (Strength = 19.1). The keywords with the longest outbreak time were tumor necrosis factor and Fas ligand, which lasted for 14 years from 2000 to 2013. In the past three years, due to COVID-19, “sars coronavirus”, “coronavirus”, and “respiratory syndrome coronavirus” have become the keywords of continuous outbreaks.

## 4 Discussion

In this study, we collected 3495 publications from the SCI-E of WoSCC from 2000 to 2022 on the role of PCD in ALI/ARDS. Using VOSviewer and CiteSpace, we analyzed the growth trend of the number of publications in this field. Our findings show that the number of annual publications has significantly increased, especially after 2018.

Among these publications, China has the largest number of publications (1965 articles), indicating that China is the most productive country in this field. However, the United States has the greatest impact in terms of total citation. Before 2005, global publications on the role of PCD in ALI/ARDS were mainly concentrated in the United States, with few publications from China and Japan. Japan's annual number of publications tended to be fixed around 2010, while China's number of publications began to show a significant growth trend. In 2018 and 2022, both China and the United States had a significant increase in the number of publications. Since the beginning of the new century, China has gradually become the country with the most publications in this field. A considerable part of this has benefited from the National Natural Science Foundation of China, which was established in 1986, and the subsequent natural science foundation of various provinces in China. The extensive development of this foundation has provided great assistance to China's scientific research activities in this field. At the same time, ARDS is a common cause of death in critically patients during the outbreak of the COVID-19. Therefore, China has invested considerable scientific research funds in related fields, which has also directly led to a sharp increase in the number of Chinese publications in this field in the past three years. In the United States and Japan, the related research in this field started earlier, and some basic and forward-looking articles were published at the beginning of the century. These earlier studies have laid the foundation for the number of citations of scientific research achievements of the two countries in this field in recent years. For instance, in the United States, the initial exploration of pyroptosis in the field of ALI, along with studies on the NLRP1-dependent pyroptosis pathway and the impact of Toll-like receptor on apoptosis, sparked great enthusiasm among scientific researchers. This resulted in a substantial number of related articles being published. Similarly, in Japan, early research on Fas/FasL-dependent apoptosis and caspase inhibitors of apoptosis in 2001 greatly inspired researchers, leading to the development of a series of related papers. The high-quality articles produced during these early years have made significant contributions to the citation count in both the United States and Japan. Overall, this study highlights the importance of PCD in ALI/ARDS research, and the need for continued efforts to promote impactful research in this field.

After investigating the current state of scientific research collaboration among countries worldwide, our findings indicates that the intensity of cooperation between China and the United States, as well as other countries, is very high. This partially explains why China and the United States can publish more articles in this field. Furthermore, by examining the average publication year of countries around the world, we have observed a gradual shift in the center of publication quantity from the United States to China from 2000 to 2022. It is possible that China has made remarkable progress while investing significant scientific research resources in this field, which aligns with the trend of China's publication output since the turn of the century. To summarize, while the United States produced influential articles in this field in the early years with a relatively stable publication rate, China has emerged as the country with the largest publication output in this field since the new century, with rapid growth in recent years.

We have shifted our research perspective from countries to specific research institutions. Among these institutions, Shanghai Jiao Tong University has been the most active in publishing articles in this field, demonstrating significant investments in scientific research and noteworthy achievements. The University of Toronto in Canada ranks low among the top ten productive institutions in terms of publication output, but its publications have the highest number of citations. This suggests that the institution produced man high-quality publications in the early years, which laid a solid foundation for the development of this field.

The cooperation map indicates that the focus of research in this field has shifted to the excellent institutions in China since 2010. Additionally, extensive cooperation has been formed between research institutions in various countries, indicating that scholars and institutions worldwide have actively contributed their expertise to eliminate academic barriers and promote academic cooperation and exchanges.

We also analyze the researchers in this field and found that Wang Ping is the most prolific author with 23 published papers. However, the author's cooperation diagram indicates relatively little collaboration with other authors. The highest level of cooperation is with Gustavo Matute-Bello. In contrast, Lee and Pi have published only six articles but have the most citations, up to 2016 times. There are many authors with similar citation rates who have published fewer papers, and their level of cooperation with other authors is significant. However, it is important to strike a balance between the number and quality of published articles in the future. While high publication rates may increase an author's productivity, it is essential to prioritize the quality of the research and the level of cooperation with others to achieve greater impact in the field.

Co-citation analysis of articles and authors is distinct from citation analysis of articles and authors. Author co-citation analysis (ACA) has gained popularity in studying the knowledge structure of academic fields and the implicit social structure of the corresponding community since White and Griffith's work in 1981 [26]. Co-cited publications refer to those cited as references concurrently with a publication and serve as the basis for further research, allowing readers to identify development trends and research hotspots in the field [27]. Articles with high co-citation times generally possess two characteristics. Firstly, they cover fundamental research, and secondly, they provide novel ideas for subsequent researchers in related fields. For instance, Ware lb's "The Acute Respiratory Distress Syndrome" and Rubenfeld gd's "Incidence and Outcomes of Acute Lung Injury" are classic research on ALI/ARDS that are widely cited by researchers in the field, confirming the characteristics of articles with high co-citation [28,29]. Future researchers interested in conducting in-depth research in this field can draw inspiration from these high co-citation publications.

The influence of a journal is closely related to the influence of its publications [30]. Among the many journals with the highest number of publications in this field, the Journal of Clinical Investigation has the highest impact factor (IF) of 19.477. As a prestigious clinical medical journal, it publishes articles on basic and phase I/II clinical research in all biomedical disciplines, including autoimmunity, immunology, pulmonary disease, vascular biology and more. The journal demands innovative research and high-quality articles and requires a close connection between basic and clinical research. Some high-quality research on the role of PCD in ALI/ARDS has been published in this journal, such as the regulation of neutrophil lifespan by inducing apoptosis in ALI, which is vital for maintaining an effective host response and preventing excessive inflammation. The American Journal of Physiology-Lung Cellular and Molecular Physiology is the most prolific journal in the field with an IF of 6.011, mainly focused on the medical respiratory system. The publications on the role of PCD in ALI/ARDS align with the journal's direction, and many researchers choose to publish their results in this journal. The role of PCD in ALI/ARDS is an emerging and developing topic that has received widespread attention in the world and has been published in various academic journals, indicating the importance of the topic.

Dual-map analysis combines macro-disciplinary fields with more specific micro-related professional research. It helps to analyze the citation trajectory of related fields through visual presentation. By looking for the subject journals with the most citations on the side of the picture, it is easy to find the subject of the scientific research field it focuses on. At the same time, following the citation path to the other side of the picture, you can find research areas that could integrate previous work in multiple disciplines to develop new work. In the process of studying the role of PCD in the occurrence and development of ALI/ARDS, researchers find that this is a work that requires multidisciplinary efforts. The study shows that basic disciplines such as immunology and molecular biology play an important role in it. For example, cell death promotes tissue homeostasis by balancing mitosis and pathology, and changes in apoptotic cells contribute to the pathogenesis of autoimmune diseases [31]. Recent studies have also shown the importance of caspase-driven pyroptosis in ALI and identified caspase-11 as an important therapeutic target [32,33]. Additionally, the role of ferroptosis in LPS-induced ALI has been inferred based on previous studies of ferroptosis in radiation-induced pulmonary fibrosis [[34], [35], [36]]. These basic studies provide inspiration for later researchers to promote the progress of basic research in this field and achieve new results in clinical transformation [[37], [38], [39]]. However, the results reveal that in the study of human ALI/ARDS, certain findings from other disciplines, such as parasitology, have been referenced, leading to significant breakthroughs. For instance, researchers have successfully reduced the severity of ALI by inhibiting PCD triggered by the caspase-linked mechanism in a mouse malaria model. This study holds the potential to enhance strategies for preventing ALI-associated mortality in malaria patients. Therefore, it is of immense significance to closely monitor the research advancements in diverse disciplines, as they contribute to the resolution of ALI/ARDS-related issues [40]. Although much progress has been made in elucidating the basic mechanisms of PCD since the turn of the century, the use of this knowledge in treatment is still in the early stages. In the future, scholars in this field will need to not only deepen their understanding of the basic mechanisms but also build a bridge to clinical practice. The research in one field is promoted by multiple disciplines. The broad vision of researchers will help them make further breakthroughs in this field, and transform the results of basic medicine into clinical practice, which will bring good news to patients.

Out of the 3495 publications in this field, we selected the top 10 most highly cited publications. Two of them focused on pyroptosis and five on apoptosis, indicating that research on these types of PCD has had the greatest impact. However, other types of PCD are still not widely studied. The most highly cited publication was published in the journal Nature Medicine, which has the highest IF of 87.244. In this paper, researchers reported on the protective effect of hyaluronic acid on apoptosis of epithelial cells, partly through the basic activation of TLR-dependent NF-κB. The interaction between hyaluronic acid-TLR2 and hyaluronic acid-TLR4 provides a signal to initiate an inflammatory response, maintain epithelial cell integrity, and promote recovery from ALI [41]. Another highly cited paper, ranked fourth in our selection, reported that systemic exposure to bacterial endotoxin lipopolysaccharide (LPS) can lead to severe endothelial cell pyroptosis, which is mediated by inflammatory caspase [32]. These highly cited papers provide valuable insights for later researchers who wish to conduct research in this field.

Since the turn of the century, research on the role of PCD in ALI/ARDS has been evolving, with changing keywords reflecting the development of each research hotpots. Over time, the focus of PCD in this field has shifted from “Apoptosis” and “Autophagy” to “Pyroptosis”, “Necroptosis” and “Ferroptosis”. In a 2008 review, Peter et al. summarized the various forms of cell death in ALI, including apoptosis and autophagy, which were hotspots of research at the time [42]. In subsequent years, many researchers focused on the role of apoptosis and autophagy in ALI/ARDS, from basic to clinical studies. For instance, in 2001, Yoshihiro Kitamura et al. confirmed the involvement of Fas L/Fas system and perforin/granzyme system, two major independent pathways of apoptosis, in the mouse model of LPS-induced ALI [43]. Additionally, studies showed that overexpression of LC3 gene can increase the clearance rate of autophagosomes and improve the survival rate in ALI mouse models [44]. However, new types of PCD have been discovered, including pyroptosis, necroptosis and ferroptosis, which have become hot research directions after 2018. In recent years, many scientists have focused on these new forms of PCD as potential ways to reduce ALI. For example, articles like “Inhibiting NLRP3-mediated pyroptosis through AMPK-dependent pathway”, “Reducing its mediated necroptosis by inhibiting RIP3”, and “Inhibiting ferroptosis through Keap1-Nrf2/HO-1 pathway” have demonstrated promising results [[45], [46], [47]].

Furthermore, despite being the oldest form of programmed cell death, apoptosis continues to be a frontier research hotspot that has been extensively explored by researchers since the last century. As shown in Fig. 10, the research hotspot within this field has shifted from the traditional pathway of apoptosis to the exploration of neutrophil extracellular traps (NETs). Neutrophils are characterized by a short lifespan and undergo caspase-dependent apoptosis within a few hours. Researchers have identified the feasibility of delaying neutrophil apoptosis as a means to inhibit immune responses, promote inflammation elimination, and restore body homeostasis [48]. Consequently, inhibiting NET formation to alleviate the progression of acute lung injury has emerged as a new research hotspot within the field. Autophagy, being an established member of programmed cell death, has experienced a resurgence of interest in recent years. Additionally, autophagy plays a key role in the process of NETs formation. Furthermore, the regulation mechanisms of PCD, such as pyroptosis, mentioned in the article, are also involved in the autophagy mechanism of macrophages. This broad involvement across various PCD regulation mechanisms has captured the attention of researchers. Consequently, an increasing number of scientific research studies have reported on the role of macrophage autophagy in the pathophysiological mechanism of ALI/ARDS [49]. Necroptosis is mainly characterized by the dissolution of cell death and the resulting endogenous inflammatory mediators. However, the regulatory pathway of necroptosis in the pathophysiological mechanism of ALI has not been elucidated. Researchers specifically focused on the life and death of mitochondria and the role of inflammatory factors represented by tumor necrosis factor in the pathophysiological mechanism of ALI/ARDS [[50], [51], [52]]. Pyroptosis is a new way of cell death in recent years. Researchers in this field have found that macrophage pyroptosis is a process in which cell death releases pro-inflammatory cytokines. Therefore, macrophage pyroptosis may partially explain the uncontrolled lung inflammation of ALI/ARDS. This signal has greatly attracted the attention of researchers, leading to a sharp rise in the study of pyroptosis in ALI/ARDS and becoming a new star in recent years [53,54]. Ferroptosis, being the most recent addition among the five PCDs, is a distinctive iron-dependent form of programmed cell death. Due to its unique cellular pathway, which relies on iron overload and lipid peroxidation, researchers believe that inhibiting ferroptosis may have the potential to ameliorate the severity of ALI/ARDS. By targeting the mechanisms involved in ferroptosis, researchers aim to explore new avenues for therapeutic interventions and potential strategies to mitigate the progression of ALI/ARDS [55].

In summary, the recent emergence of hotspots in new types of PCD is highly promising. In this complex game of PCD, various core players can disrupt the fragile balance of the cellular environment, leading to either life or death, and the shift from pro-inflammatory to anti-inflammatory signals. Consequently, an increasing number of researchers are investing in this field with the aim of regulating PCD to treat ALI/ARDS.

When discussing the role of programmed cell death in ALI/ARDS, COVID-19 inevitable becomes a topic of interest in the past three years. Keywords such as “SARS coronavirus”, “coronavirus”, and “respiratory syndrome coronavirus” have gained prominence in ongoing surveillance efforts. SARS-CoV-2, the virus responsible for COVID-19, is a cytopathic virus that induces cell death during viral replication. Virus replication in epithelial cells may also lead to high levels of pyroptosis, an inflammatory form of PCD observed in cytopathic virus infection [56,57]. On the other hand, damaged cells undergo apoptotic cell death during infection, inhibiting viral replication [58]. Some viruses can prevent apoptosis by inhibiting caspase-8 [59]. Necroptosis plays a role in cell death after viral infection, but whether it is beneficial or detrimental to the host antiviral response or tissue inflammation in COVID-19 remains uncertain. Autophagy acts as a cell surveillance mechanism against invading pathogens [60]. Autophagy can degrade lysosomes by delivering viruses or viral proteins, activate innate and adaptive immune responses by transporting viral nucleic acids and antigens to the lysosome compartment, and act as a defense system by regulating virus-induced cell death [[61], [62], [63]]. Currently, experiments have shown that the endocytosis pathway of autophagy plays a key role in mediating the entry of many coronaviruses, including SARS-CoV, MERS-CoV and possibly SARS-CoV-2. Strategies for inhibiting lipid peroxidation have become an attractive cell protection method due to the key role of lipid peroxidation in ferroptosis and the possible contribution of ferroptosis to COVID-19 [38]. Intracellular iron capture to remove GSH, inactivate GPX4 and up-regulate PUFA peroxidation are components of SARS-CoV-2 infection and ferroptosis, indicating that ferroptosis plays an irreplaceable role in the pathogenesis of COVID-19 organ damage [64,65]. COVID-19 mainly attacks the patient's lung respiratory system and causes systemic organ damage. At present, the academic community believes that COVID-19 can easily cause ALI and then progress to ARDS, causing a series of disorders in the patient's health system and even death. Therefore, the outbreak of COVID-19 has had a significant impact on the research of ALI/ARDS in various aspects. It has not only prompted investigations into the pathophysiological mechanisms underlying ALI/ARDS but also spurred efforts to identify small biological molecules that can serve as indicators for monitoring the severity of ALI. Furthermore, there is an urgent need to develop drugs that can effectively slow down the progression of ALI by targeting its underlying mechanisms. As a result, the number of articles published by research institutions around the world has increased rapidly in the past three years. In such an external environment, the study of the role of PCD in ALI/ARDS holds great potential for advancing human health. However, it also brings considerable pressure and challenges.

These articles not only reveal the different mechanisms of ferroptosis in ALI/ARDS but also conduct in-depth research on the interconnection between different types of PCD. The role of PCD in ALI/ARDS in the future still has broad research prospects. Translating the research results in this field into clinical application to improve the prognosis of ALI/ARDS is the future research direction in this field. The analysis of keywords in this field may provide inspiration for future researchers who intend to engage in this field and help them find research hotspots and directions.

Although since the new century, bibliometrics has been widely used in biology, medicine and other fields. However, the bibliometric research on ALI/ARDS is not sufficient. Such as the analysis of the overall study of ALI/ARDS in the past years [66]. Specific areas include discussion of macrophages, NLRP3 inflammasome, and mitochondria [[67], [68], [69]]. However, the role of PCD in ALI/ARDS has not been systematically studied. Through the analysis method of bibliometrics, this study shows that the field has broad development prospects and is in a booming trend. We analyze the influence of countries, research institutions and authors on this field, and summarize the changes of research hotspots in this field since the new century. Moreover, the impact of the COVID-19 epidemic in the past three years on this field was analyzed.

The field of ALI/ARDS has witnessed significant transformations in the understanding of PCD over the years. Initially focused on apoptosis and autophagy, it has now expanded to include newly discovered forms of PCD such as pyroptosis, ferroptosis, and necroptosis. However, the significance of the older types of PCD has not been disregarded, and continued research has shed new light on their relevance. The emergence of cross-cutting areas and major health incidents, such as the COVID-19 pandemic, has also influenced the field and its research priorities.

As a result, the future research hotspots within this field are expected to be distributed among the following sub-areas: PCD pathways involved in COVID-19 and their impact on disrupting the homeostasis of the human respiratory system; The regulatory role of lncRNAs and microRNAs in PCD processes; NETosis, a recently identified form of apoptosis, and its implications in the field; The impact of mitochondrial necroptosis on the progression of ALI/ARDS; Exploring the relationship between pyroptosis and macrophage autophagy; Investigating the role of ferroptosis in ALI/ARDS. By focusing on these sub-areas, researchers can contribute to the advancement of knowledge and understanding in the field of PCD in ALI/ARDS and address the emerging challenges and opportunities within these specific research domains.

## 5 Limitation

It is important to note that limitations in data sources, publication languages and formats may have excluded some publications in this field. Additionally, novel types of PCD such as cuproptosis were not included in the analysis, which may have contributed to the limitations of the study. However, overall, this study provides sufficient analysis of the literature in this field to provide reference value for researchers.

## Author contribution statement

Lihong Wang, Ph.D.: Conceived and designed the experiments.

Enyao Huang: Performed the experiments; Analyzed and interpreted the data; Wrote the paper.

Li Gao; Ruiyu Yu: Performed the experiments; Analyzed and interpreted the data.

Keying Xu: Contributed reagents, materials, analysis tools or data.

## Funding Statement

This study was supported by the National Natural Science Foundation of China (Grant No.81972478), the Jiangsu Provincial Key Laboratory of Critical Care Medicine (JSKLCCM-2022-02-013), and the Student Research Training Program of Jiangsu Province (202210286232Y).

## Data availability statement

Data will be made available on request.

## Declaration of competing interest

The authors declare that they have no known competing financial interests or personal relationships that could have appeared to influence the work reported in this paper.
